# Infectious Disease Containment Based on a Wireless Sensor System

**DOI:** 10.1109/ACCESS.2016.2551199

**Published:** 2016-04-06

**Authors:** Xiao Sun, Zongqing Lu, Xiaomei Zhang, Marcel Salathé, Guohong Cao

**Affiliations:** 1 Department of Computer Science and EngineeringThe Pennsylvania State University University ParkPA16802USA; 2 Department of BiologyThe Pennsylvania State University University ParkPA16802USA; 3 School of Life Sciences and School of Computer and Communication SciencesÉcole Polytechnique Fédérale de Lausanne Lausanne1015Switzerland

**Keywords:** Wireless sensor system, human contact, node centrality, disease containment

## Abstract

Infectious diseases pose a serious threat to public health due to its high infectivity and potentially high mortality. One of the most effective ways to protect people from being infected by these diseases is through vaccination. However, due to various resource constraints, vaccinating all the people in a community is not practical. Therefore, targeted vaccination, which vaccinates a small group of people, is an alternative approach to contain infectious diseases. Since many infectious diseases spread among people by droplet transmission within a certain range, we deploy a wireless sensor system in a high school to collect contacts happened within the disease transmission distance. Based on the collected traces, a graph is constructed to model the disease propagation, and a new metric (called connectivity centrality) is presented to find the important nodes in the constructed graph for disease containment. Connectivity centrality considers both a node’s local and global effect to measure its importance in disease propagation. Centrality based algorithms are presented and further enhanced by exploiting the information of the known infected nodes, which can be detected during targeted vaccination. Simulation results show that our algorithms can effectively contain infectious diseases and outperform other schemes under various conditions.

## I. Introduction

Infectious diseases pose a serious threat to public health due to its high infectivity and potentially high mortality. Over the last few decades, infectious diseases have caused several regional and worldwide pandemics, resulting in many infections and deaths. For example, during H1N1 pandemic in 2009, more than 600,000 cases were lab-confirmed and more than 14,000 were dead all over the world [1]. According to World Health Organization (WHO), the epidemic of Ebola in 2014 has caused significant mortality in many West African countries, with a reported case with fatality rate of 70% [2]. Even for a seasonal influenza, it is estimated to affect 5% to 15% of the global population and cause 3 to 5 million cases of severe infections and 250,000 to 500,000 deaths worldwide each year [3].

To prevent infectious diseases, one of the most effective ways is to vaccinate the susceptible individuals. However, due to resource constraints such as the limited vaccine supply, in many cases, it may not be practical to vaccinate all the susceptible individuals, especially when a new infectious disease outbreaks. Therefore, *targeted vaccination*, which vaccinates a small group of people in a community, is an alternative approach to contain infectious diseases. The challenge is how to find the group of people whose vaccination will averagely result in the maximum reduction of disease spread.

**FIGURE 1.**
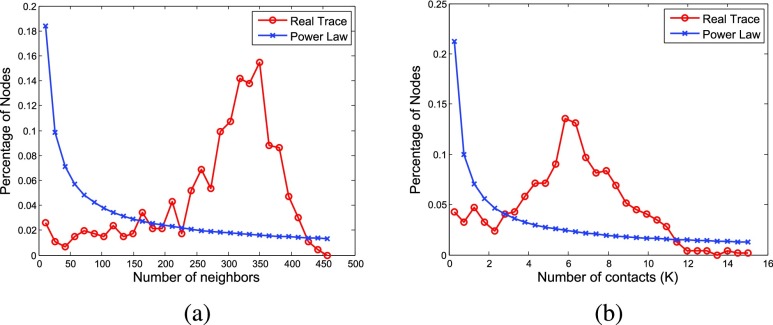
Distributions of the number of nodes’ (a) neighbors and (b) contacts.

Targeted vaccination has been studied in some previous works [4]–[7]. However, these works are limited to theoretical analysis based on synthetic networks such as random, homogeneous or scale-free network, which may not reflect the real contact patterns among people in different scenarios. For example, based on the contact traces collected from high school students, where each student carries a sensor node to record the contacts with others by sending and receiving packets (details shown in Section III), we show in Figure 1 that the distributions of the number of nodes’ contacts (i.e., the number of packets received from other nodes) and neighbors (i.e., the number of nodes from which packets are received) are different from the power-law distributions. Since students in the high school spend most of their time in classes and students in the same class may have contacts with each other, most of the nodes in the network will have similar number of neighbors and contacts. As observed from our collected trace, the number of nodes’ neighbors and the number of nodes’ contacts are more likely to follow normal distributions rather than power-law distributions.

The problem of targeted vaccination has some similarity to virus (worm) containment in the area of computer networks, such as cellular networks [8]–[10] or online social networks [11]–[14]. Based on cluster partition and community detection, different schemes have been proposed for virus (worm) containment [8], [11]. By using various techniques to divide the network into different partitions, these schemes contain the viruses (worms) within the infected partition before they spread out. More specifically, the nodes that separate network partitions are vaccinated in [8] and the neighbors of overlapped nodes between two communities are vaccinated in [11]. However, these works mainly focus on containing the worm (virus) in cellular network or online social network, which has different propagation patterns from infectious disease. For infectious disease, it will be transmitted with a probability when two people contact with each other. However, in cellular network or online social network, a node will be infected immediately once it contacts with an infected node. In addition, they implicitly assume that all nodes in the network are eligible for vaccination, which is not true in disease containment (e.g., we cannot vaccinate an infected node). Thus, we cannot directly apply these schemes to targeted vaccination.

Different from the aforementioned works, we deploy a wireless sensor system in a high school to collect contacts among students. Since the wireless signal strength degrades as the communication distance increases [15], we can measure the wireless signal strength and then infer when and where students meet with each other. This information is important for modeling the propagation of infectious disease. Many respiratory infectious diseases (e.g., influenza) spread among people by droplet transmission, requiring an infected and a susceptible person to be in close physical contact at a short maximum distance [16]–[18]. With our wireless sensor system, we can find student contacts within such distance, and construct a *disease propagation graph* to model the infectious disease propagation. Then, targeted vaccination becomes a problem of selecting important nodes in a graph to contain infectious disease.

Based on the disease propagation graph, node centrality can be used to measure its importance during disease propagation. Although there are some centrality measures [19] such as degree centrality, betweenness centrality and closeness centrality, they all have disadvantages when applied to disease containment. For example, degree centrality only considers the connection between a node and its neighbors, and thus is limited by its local effect. Betweenness centrality measures the global effect, but it does not describe the difference between a node’s influence on its neighbors and that on those nodes far away. Closeness centrality does not work when the graph is disconnected. In disease propagation, an infected node will infect nodes closer with much higher possibility than those far away. Thus, we propose a new metric called *connectivity centrality*, which takes into account a node’s influence on all others and considers nodes closer more important. Based on the proposed centrality measure, we design centrality based algorithm for targeted vaccination. In infectious disease containment, not all nodes are eligible for vaccination. For example, vaccination for a node which has already been infected will not be effective. Some of these infected nodes can be detected during vaccination. With this information, we enhance the centrality based algorithm by considering both a node’s infecting capability and its infected possibility. We evaluate the centrality based algorithm and the enhanced algorithm, and compare them with other schemes. The trace driven simulation results show that our algorithms can significantly reduce the infection rate. Although our algorithms are illustrated based on the contact trace collected from a high school, they are trace-independent and can work in other networks.

The rest of this paper is organized as follows. Section II reviews related work and Section III describes our trace collection. We propose centrality based algorithms and enhanced algorithm in Section IV and evaluate the performance in Section V. Section VI concludes the paper. A preliminary work has been published in [20].

## II. Related Work

A rich body of work has focused on infectious disease containment. Various disease propagation patterns have been studied in [21] and [22]. Based on the disease propagation model, Prakash *et al.* [21] derived the epidemic threshold for a given network under which an epidemic will not happen and above which an epidemic will happen. Moreover, they have designed a greedy strategy, which vaccinates the node that causes the largest drop in the eigenvalue of the system matrix. Cohen *et al.* [22] proposed a mathematical model and an immunization policy based on a small fraction of random acquaintances, and analytically studied the critical threshold for complete immunization. However, these techniques mainly focus on how to avoid the spreading of infectious disease becoming an epidemic, without considering how to decrease the number of infected individuals in a community.

With using the SIR (Susceptible-Infected-Removed) epidemiological model, Madar *et al.* [23] studied the epidemic spreading behavior in scale-free networks and proposed different immunization strategies. In [24], Hayashi *et al.* investigated the spread of viruses in growing scale-free networks with new users coming, and compared the performance of targeted vaccination and random vaccination under such network models. However, in these works, they assume that the graph is scale-free and the connections between nodes follow power law distribution.

By analyzing a real cellular network trace, Zhu *et al.* [8] constructed a graph to describe the social relationships between mobile phones, and proposed two algorithms (balanced partitioning and cluster partitioning) to contain mobile worms at the early stage. Nguyen *et al.* [11] utilized community structures to contain viruses in online social networks. They presented community detection algorithms to find the overlapping communities and patched the nodes in the overlapped areas to prevent worms spreading from one community to another. With the community structure, Lu *et al.* [12] calculated the intra-centrality (within community) and inter-centrality (between community) and combined them together to select nodes for vaccination. The Facebook trace in New Orleans regional network was used in [11] and [12]. However, these works mainly focus on the worms (viruses) in cellular networks or online social networks, which have different propagation patterns from infectious diseases. In addition, they implicitly assume that any node in the network can be selected as vaccinated node, while in disease containment, this is not true (e.g., we cannot vaccinate a node that has already been infected).

This paper extends the preliminary version of our algorithm appeared in [20]. In [20], we proposed connectivity centrality and designed an algorithm based on the proposed centrality. In this paper, we enhance the centrality based algorithm by exploiting information of the known infected nodes which can be detected during targeted vaccination. Given a set of known infected nodes, we measure node’s infecting capability and its possibility to be infected, and consider these two factors to select nodes to be vaccinated.

## III. Trace Collection

Most infectious diseases spread among people through virus, which is transmitted by airborne infectious particles or small respiratory droplets when two people contact within a certain distance [16]–[18]. Besides, the activity of many infectious viruses (e.g., influenza virus) varies in indoor and outdoor environment because of the different ambient airflow patterns [25], [26]. Therefore, collecting the contacts among people within the disease transmission distance and indicating whether a contact happened indoor or outdoor are important for modeling disease propagation and designing disease containment algorithms. However, most of the existing traces do not consider these two factors, and thus we deploy a wireless sensor system in a high school and collect our own traces.

### A. System Overview

**FIGURE 2.**
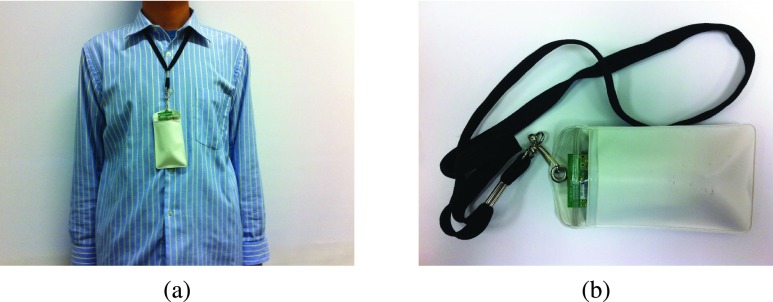
A mobile mote carried by a student.

Due to the frequent and close contacts among students every day, schools are regarded to play a major role in the spread of infectious diseases into the community [27], [28]. Therefore, we deployed our trace collection system in a high school which has about 800 students. The Crossbow TelosB mote, which has a low-power microcontroller, an IEEE 802.15.4 radio and extended memory, is used to collect student contacts. Since the wireless signal strength degrades as the communication distance increases, we can measure the wireless signal strength and then infer when and where students meet with each other. In the wireless sensor system, we have two types of motes: *mobile motes* and *stationary motes*. Mobile motes are carried by students to collect their contacts. As shown in Figure 2, each mobile mote is placed in a pouch attached to a lanyard and worn by a student around his (her) neck. During a school day, the mobile motes are carried by students and each of them is labeled with a unique ID. The mobile mote broadcasts a beacon every 20 seconds and keeps listening to the wireless channel to record beacons from other motes. The beacon includes mote type, mote ID, and its local sequence number which is initialized to 0 and increased by one after each beacon broadcast. Stationary motes are deployed at some fixed places (e.g., classrooms, dining halls and restrooms) to indicate the contact locations. Each stationary mote is also assigned a unique ID and broadcasts beacons with its mote type, ID and sequence number at an interval of 20 seconds. The sequence number starts at 0 when the mote is powered on and increased by one after each broadcast. During trace collection, all the motes keep broadcasting beacons periodically and only mobile motes record beacons from others. Beacons from other mobile motes are recorded as contact information and beacons from stationary motes are recorded to infer whether the contacts happen indoor or outdoor.

The wireless sensor system is deployed during a flu season in 2012. On each school day, the mobile motes are distributed to students around 7 am and received back around 4 pm. In order not to disturb students’ activities, the stationary motes are deployed at night before the trace collection and their starting times are recorded manually. The experiment was conducted across two weeks in March 2012. Averagely, 3.4 million contacts were collected between mobile motes each day.

### B. Design Considerations

#### 1) Disease Transmission Distance

According to [17], [18], and [29], the airborne droplets can only transmit from one person to another when their contact distance is less than 9 feet. Thus, 9 feet is a critical distance for disease propagation and we only need to collect contacts within this distance. By using received signal strength indicator (RSSI), which reflects the distance between the sending and receiving nodes, we can determine if a contact happens within a specific range by checking if the corresponding RSSI is above certain threshold for a given transmission power.

Different from many existing sensor network applications [30]–[33], where wireless nodes are supposed to communicate with each other with the highest transmission power to achieve higher data delivery rate and reach larger coverage area, we choose a lower transmission power in our system to save energy. Since a TelosB mote only has two AA batteries as its power supply, if it keeps working at the highest transmission power level, its batteries will die very quickly. According to our preliminary experimental results, the transmission power of −16.9 dBm (power level 6 for TelosB mote) is strong enough to ensure a high data delivery rate within a distance of 9 feet. Under this transmission power, the RSSI of the packet received within 9 feet is larger than −80 dBm. Therefore, in our implementation, the transmission power of the mobile mote is set to −16.9 dBm and a beacon from the mobile mote is recorded only if its RSSI is larger than −80 dBm.

#### 2) Indoor or Outdoor

Since the infecting capability of infectious disease varies in indoor and outdoor environment due to the different ambient airflow patterns [25], [26], stationary motes are deployed to provide location information for inferring where a contact happens. In our system, stationary motes periodically broadcast beacons with transmission power of −11 dBm (power level 10 for TelosB mote) and they are carefully deployed to cover the entire buildings in the school. Thus, if a mote is indoor at some time, it will receive beacons from at least one stationary mote at that time. Further, if a beacon is received from a mobile mote and at the same time both the sender and receiver have recorded beacons from some stationary motes, we can infer that this contact happens indoor; otherwise, it happens outdoor. Therefore, we can discern whether a contact happens indoor or outdoor by checking beacons received from the stationary motes.

## IV. Targeted Vaccination

In this section, we first construct a graph to model disease propagation based on the collected traces. Then, we propose centrality based algorithm for disease containment, and further enhance the solution by exploiting the knowledge of the infected nodes which have been detected during vaccination.

### A. Disease Propagation Graph

If there is a contact between two students, there will be some probability for the infectious disease to be transmitted between them. Therefore, we can construct a graph (called *disease propagation graph*) to model disease propagation based on the collected human contacts. The disease propagation graph is represented by $G = (V,E)$, where $V$ is the set of vertices and $E$ is the set of edges. In graph $G$, each node $u \in V$ represents a participant and an edge $e = (u,v) \in E$ exists when there is contact between $u$ and $v$. Since the infectious disease is transmitted bidirectional, $G$ is an undirected graph.

In the disease propagation graph, we assign each edge $(u,v)$ a weight $w(u,v)$ to describe the disease propagation probability between these two nodes. Two factors should be considered when assigning the edge weight: contact frequency and contact location. For two nodes that contact with each other frequently (i.e., they spend a lot of time together), if one node gets some infectious disease, the other one is most likely to be infected. Thus, the more frequently two nodes encounter, the larger weight should be assigned to the corresponding edge. Another factor that affects the probability of infection is contact location. According to [25] and [26], infectious disease such as influenza, is more likely to spread quickly in indoor environment than outdoor environment. Thus, contacts happen indoor should be assigned more weight than contacts happen outdoor.

Considering both contact frequency and contact location, the edge weight $w(u,v)$ is calculated as:

\begin{equation*} w(u,v) = \frac {\sum _{t=0}^{T} r(u,v,t)\eta (u,v,t)}{T} \end{equation*}

where

\begin{align*} r(u,v,t)=&\begin{cases} 1 & \textrm {if there is a contact between $u$ and $v$ at $t$;}\\ 0 & \textrm {otherwise.} \end{cases}\\ \eta (u,v,t)=&\begin{cases} 1 & \textrm {if the contact between $u$ and $v$ at} \\ & \textrm {time $t$ happens indoor;} \\ \eta _{0} & \textrm {otherwise.} \end{cases} \end{align*}

and $T$ is the time period of the trace used for constructing the graph.

In our trace collection system, each mote (either mobile mote or stationary mote) periodically broadcasts a beacon whose local sequence number is initialized to 0 and increased by one after each broadcast. Since there are many stationary motes whose starting times are manually recorded, beacons received from these motes can be used to synchronize local sequence numbers in the beacons received from mobile motes. Therefore, we use the synchronized global sequence number to represent time $t$. $r(u,v,t)$ is set to 1 when $u$ receives a beacon from $v$ at $t$ or $v$ receives a beacon from $u$ at $t$. $\eta (u,v,t)$ is set to $\eta _{0}$ if the contact happens outdoor. Since infectious disease is relatively inactive in outdoor environment, $0 < \eta _{0} < 1$ and its value depends on the characteristic of the specific disease.

### B. Centrality Based Targeted Vaccination

The disease propagation graph shows how each node contacts with others and how disease propagates among them. In the graph, each node has different influence on others and thus plays a different role during disease propagation. Since the importance of each node on disease propagation can be measured by centrality, we propose centrality based algorithm for targeted vaccination.

In literature, there are some well known centrality metrics [19] such as degree, betweenness and closeness centrality.

**Degree centrality** measures how well a node is connected with its neighbors and it is defined as:

\begin{equation*} C_{d}(u) = \sum _{v\in N(u)}w(u,v) \end{equation*}

where $N(u)$ is the set of $u$’s neighboring nodes.

**Betweenness centrality** measures to what extent a node can connect two other nodes through a shortest path and it is defined as:

\begin{equation*} C_{b}(u) = \sum _{s\neq u\neq t, s, t \in V} \frac {\sigma _{st}(u)}{\sigma _{st}} \end{equation*}

where $\sigma _{st}$ is the total number of shortest paths from node $s$ to $t$ and $\sigma _{st}(u)$ is the total number of shortest paths from node $s$ to $t$ that go through node $u$.

**Closeness centrality** measures how close a node is to others and it is defined as:

\begin{equation*} C_{c}(u) = \frac {|V|-1}{\sum \limits _{v \neq u, v \in V} d(u,v)} \end{equation*}

where $|V|$ is the cardinality of $V$ and $d(u,v)$ is the shortest path distance between $u$ and $v$.

**FIGURE 3.**
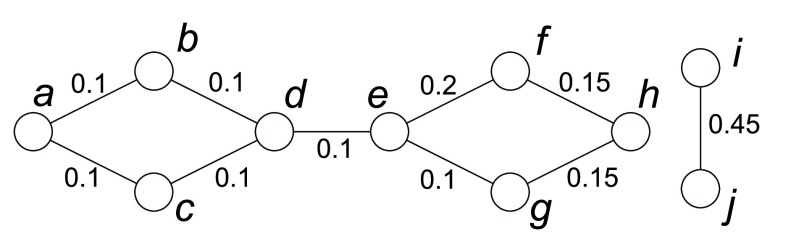
An example of different centrality metrics in the disease propagation graph. The edge weight $w(u,v)$ is shown in the graph and $\frac {1}{w(u,v)}$ is used as the distance between two neighboring nodes $u$ and $v$ when calculating distance based centralities. Both node $i$ and $j$ have the highest degree centrality of 0.45; node $d$ has the highest betweenness centrality of 25; all the nodes have the same closeness centrality of 0; node $e$ has the highest connectivity centrality of 0.504.

Although these centralities can be used to measure the node’s importance in a graph, they are not applicable to describe a node’s influence on others during disease propagation. For example, Figure 3 shows an example of using different centralities to remove one node to contain the disease. Both betweenness and closeness centralities are distance based and the weight between any two neighboring nodes should represent their distance. However, in the disease propagation graph, the edge weight is assigned based on the disease propagation probability. The larger the edge weight is, the closer the nodes are and the smaller their distance is. Therefore, when calculating distance based centralities, $\frac {1}{w(u,v)}$ is used as the distance between two neighboring nodes $u$ and $v$. As shown in Figure 3, both node $i$ and $j$ have the highest degree centrality; node $d$ has the highest betweenness centrality; all the nodes have the same closeness centrality of 0. However, none of these centralities returns the optimal vaccinated node $e$, whose removal will not only separate the graph into different parts, but also remove edges with large edge weights. This is because degree centrality only considers the connection between a node and its neighbors, and thus is limited by its local effect; betweenness centrality considers the global effect, but it does not describe the difference between a node’s influence on its neighbors and that on those nodes far away; with considering the distance between two nodes, closeness centrality treats a node’s influence on others differently, but its value is dominated by the path with longer distance since all the distances are simply added together, and it does not work in a disconnected graph.

In disease propagation graph, infectious disease is more likely to be transmitted to nodes closer than nodes further away. Thus we propose a new centrality metric called *connectivity centrality* to measure how contagious an infected node is to others and it is defined as:

\begin{equation*} C_{con}(u) = \sum \limits _{v \neq u, v \in V}c(u,v) \end{equation*}

where

\begin{equation*} c(u,v) = \begin{cases} \displaystyle \frac {1}{d(u,v)h(u,v)} & \textrm {if there is a path from $u$ to $v$;}\\ 0 & \textrm {otherwise.} \end{cases} \end{equation*}

and $h(u,v)$ denotes the number of hops between $u$ and $v$ along the shortest path.

Connectivity centrality takes into account a node’s effect on others and considers nodes closer more important, and thus it is better than other centrality metrics for measuring node’s influence on disease propagation. For example, in Figure 3, the optimal vaccinated node $e$ is the node with the highest connectivity centrality.

Based on node centrality, we can propose a straightforward algorithm. To find $k$ vaccinated nodes, we sort all the nodes based on their centrality values and choose the top $k$ nodes. However, in disease containment, not all nodes are eligible for vaccination. For example, vaccinating a node which has already been infected will not be effective. In addition, some nodes may refuse to get vaccinated because of their concerns on potential side effects [34]. Therefore, in the centrality based algorithm, after sorting, we select the first $k$ nodes which are eligible for vaccination as the targeted nodes.

### C. Enhanced Targeted Vaccination

**FIGURE 4.**
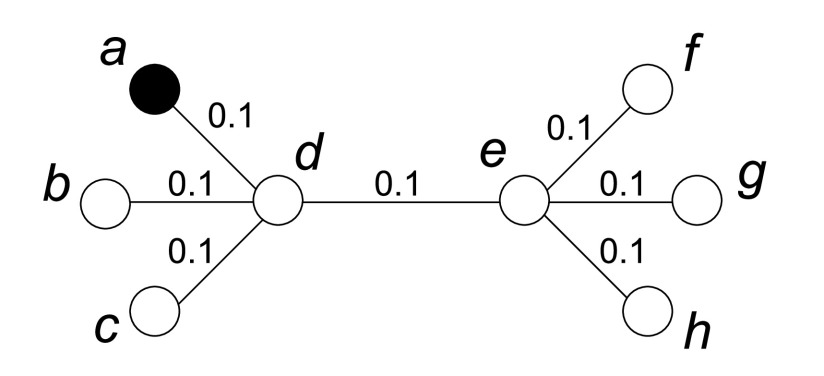
Targeted vaccination with some known infected nodes.

Centrality based algorithm selects the nodes with the highest influence to be vaccinated. This is because once these nodes are infected, they are able to infect more nodes due to their close connections. However, it only considers the infecting capability of a node, without considering how possible it will be infected. Both these two factors should be taken into account for vaccination. For example, as shown in Figure 4, each edge has the same weight and $a$ is known as an infected node. By using centrality based algorithm, both $d$ and $e$ can be selected as the candidate nodes due to their high centrality. However, since $a$ has already been infected, $d$ will be a better choice because it is more likely to be infected soon, and vaccinating $d$ will potentially protect more nodes from being infected. In order to describe both a node’s infecting capability and its infected possibility, we propose *infecting score* and *infected score* by exploiting the information of the infected nodes which can be detected during vaccination, and combine these two scores together to determine which nodes should be vaccinated.

#### 1) Infecting Score

**FIGURE 5.**
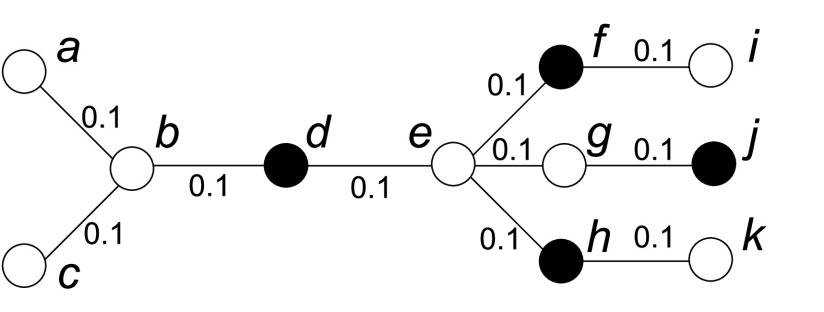
Node’s infecting capability under a set of known infected nodes. Node $e$ has the highest degree, betweenness, closeness and connectivity centrality ($C_{d}(e) = 0.4$, $C_{b}(e) = 72$, $C_{c}(e) = 0.056$ and $C_{con}(e) = 0.522$), but node $b$, which has the highest infecting score under $I=\{d, f, h, j\}$ ($\varphi (b,I) = 0.2$), is a better choice for vaccination.

When centrality is used to measure node’s influence on others, it implicitly assumes that all the other nodes are uninfected. However, if there are some known infected nodes in the graph, the calculated centrality may not accurately measure node’s importance. For example, as shown in Figure 5, the black nodes are infected nodes. With centrality based algorithm, node $e$ should be chosen to be vaccinated first since $e$ has the highest centrality no matter which centrality metric is used. However, considering that node $d$, $f$, $h$ and $j$ have already been infected, node $b$ will be a better choice since its removal will separate node $a$ and $c$ from the infected nodes, while $e$’s removal will still leave infected nodes in all partitions.

Let $I$ denote the set of known infected nodes; e.g., $I=\{d, f, h, j\}$ in Figure 5. To measure node $b$’s influence under the infected node set $I$, nodes in $I$ should not be considered since these nodes have already been infected and $b$ has no influence on them. Also, $b$ has no influence on the nodes that are closer to an infected node than to $b$. For example, even if node $b$ is infected, it will not affect node $i$’s infection status which only depends on the connection between $i$ and $f$. As a result, node $b$’s influence should be different with the knowledge of the infected nodes ($f$ in this example). Thus, we define *infecting score* to measure the infecting capability of a node under infected node set $I$. As illustrated in Section IV-B, connectivity centrality describes the local and global effects of a node, which is better than other centralities in measuring its importance during disease propagation. Based on connectivity centrality, node $u$’s infecting score under infected node set $I$ is defined as follows:

\begin{equation*} \varphi (u,I) = \sum \limits _{v \in S(u,I)} c(u,v) \end{equation*}

where

\begin{equation*} S(u,I) = \{v \mid c(u,v) \geq \max \limits _{w \in I} c(w,v), v \in V \setminus \{u\}\} \end{equation*}

Based on this definition, $\varphi (u,I) = C_{con}(u)$ when $I = \emptyset $, i.e., connectivity centrality is a special case for calculating the infecting score when no node is infected.

#### 2) Infected Score

To measure the importance of a node more accurately, besides infecting score, *infected score* is introduced to measure the possibility for it to be infected, which is calculated as follows:

\begin{equation*} \psi (u,I) = \begin{cases} 1 & \textrm {if $u \in I$;}\\ \sum \limits _{v \in N(u)}\psi (v,I) \cdot \displaystyle \frac {w(u,v)}{\max \limits _{w \in V} {C_{d}(w)}} & \textrm {if $u \notin I$.} \end{cases} \end{equation*}

**FIGURE 6.**
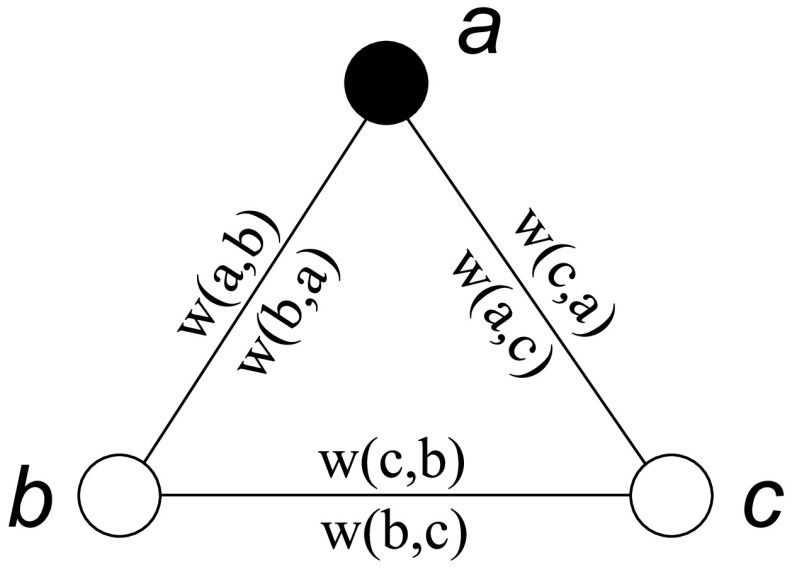
Infected score calculation in a simple graph.

Figure 6 illustrates how to apply the above equation to a simple graph, which contains three nodes $a$, $b$ and $c$. Suppose $I= \{a\}$; i.e., node $a$ has been detected as an infected node. By applying Equation 1, we have the following linear equations:

\begin{align*} \begin{cases} \psi (a,I) = 1\\ \psi (b,I) = \psi (a,I) \cdot \displaystyle \frac {w(a,b)}{W} + \psi (c,I) \cdot \displaystyle \frac {w(c,b)}{W}\\[0.4pc] \psi (c,I) = \psi (a,I) \cdot \displaystyle \frac {w(a,c)}{W} + \psi (b,I) \cdot \displaystyle \frac {w(b,c)}{W} \end{cases} \end{align*}

where $W = \max \{C_{d}(a), C_{d}(b), C_{d}(c)\}$.

In the disease propagation graph, for two nodes $u$ and $v$, we have $w(u,v) = w(v,u)$. Thus, Equation 2 can be easily solved as follows:

\begin{align*} \begin{cases} \psi (a,I) = 1\\ \psi (b,I) = \displaystyle \frac {w(a,b)W+w(a,c)w(b,c)}{W^{2}-[w(b,c)]^{2}}\\[0.5pc] \psi (c,I) = \displaystyle \frac {w(a,c)W+w(a,b)w(b,c)}{W^{2}-[w(b,c)]^{2}} \end{cases} \end{align*}

For a disease propagation graph $G = (V,E)$ with edge weight $w(u,v)$, we can calculate infected score for each node $u \in V$ by applying Equation 1. In this way, a $|V| \times |V|$ linear equation system can be generated.

Suppose the node set $V = \{u_{1}, u_{2}, u_{3}, \ldots , u_{|V|}\}$, the infected set $I = \{u_{p_{1}}, u_{p_{2}}, u_{p_{3}}, \ldots , u_{p_{|I|}}\}$ and the uninfected set $V\setminus I = \{u_{q_{1}}, u_{q_{2}}, u_{q_{3}}, \ldots , u_{q_{|V\setminus I|}} \}$. For simplicity, we denote $\psi (u,I)$ as $\psi (u)$ and denote normalized weight $\frac {w(u,v)}{\max \limits _{w \in V}C_{d}(w)}$ as $w'(u,v)$. By applying Equation 1 for each node, we have the following equations:

\begin{equation*} \begin{cases} \psi (u_{p_{1}}) = 1\\ \qquad \qquad \vdots \\ \psi (u_{p_{|I|}}) = 1\\ \psi (u_{q_{1}}) = \psi (u_{p_{1}})w'(u_{p_{1}}, u_{q_{1}}) \\ \quad ~\qquad \quad +\!\cdots \!+\! \psi (u_{p_{|I|}})w'(u_{p_{|I|}}, u_{q_{1}}) \!+\! \psi (u_{q_{2}})w'(u_{q_{2}}, u_{q_{1}}\!) \\ \quad ~\qquad \quad +\, \cdots + \psi (u_{q_{|V\setminus I|}})w'(u_{q_{|V\setminus I|}}, u_{q_{1}})\\ \qquad \qquad \qquad \quad \vdots \\ \psi (u_{q_{|V\setminus I|}}) = \psi (u_{p_{1}})w'(u_{p_{1}}, u_{q_{|V\setminus I|}}) \\ \qquad \qquad \qquad + \cdots +\, \psi (u_{p_{|I|}})w'(u_{p_{|I|}}, u_{q_{|V\setminus I|}}) \\ \qquad \qquad \qquad +\, \psi (u_{q_{1}})w'(u_{q_{1}}, u_{q_{|V\setminus I|}}) \\ \qquad \qquad \qquad +\, \cdots + \psi (u_{q_{|V\setminus I|-1}})w'(u_{q_{|V\setminus I|-1}}, u_{q_{|V\setminus I|}}) \end{cases} \end{equation*}

It can be easily transformed to a linear equation system denoted by matrices:

\begin{equation*} \left [{\begin{array}{c c} I_{|I| \times |I|} & O_{|I| \times |V\setminus I|}\\ A_{1_{|V\setminus I| \times |I|}} & A_{2_{|V\setminus I| \times |V\setminus I|}} \end{array}}\right ] \cdot \left [{\begin{array}{c} X_{1}\\ X_{2} \end{array}}\right ] = \left [{\begin{array}{c} b_{1}\\ b_{2} \end{array}}\right ] \end{equation*}

where $I_{|I| \times |I|}$ is an identity matrix, $O_{|I| \times |V\setminus I|}$ is a zero matrix,

\begin{align*} A_{1}=&\left [{\begin{array}{ccc} w'(u_{p_{1}}, u_{q_{1}}) & \cdots & w'(u_{p_{|I|}}, u_{q_{1}})\\ w'(u_{p_{1}}, u_{q_{2}}) & \cdots & w'(u_{p_{|I|}}, u_{q_{2}})\\ \vdots & \vdots & \vdots \\ w'(u_{p_{1}}, u_{q_{|V\setminus I|}}) & \cdots & w'(u_{p_{|I|}}, u_{q_{|V\setminus I|}})\\ \end{array}}\right ]\!,\\ A_{2}=&\left [{\begin{array}{ccc} -1 & \cdots & w'(u_{q_{|V\setminus I|}}, u_{q_{1}})\\ w'(u_{q_{1}}, u_{q_{2}}) & \cdots & w'(u_{q_{|V\setminus I|}}, u_{q_{2}})\\ \vdots & \vdots & \vdots \\ w'(u_{q_{1}}, u_{q_{|V\setminus I|}}) & \cdots & -1\\ \end{array}}\right ]\!,\\ X_{1}=&\left [{\begin{array}{c} \psi (u_{p_{1}})\\ \vdots \\ \psi (u_{p_{|I|}}) \end{array}}\right ]\!, \quad X_{2} = \left [{\begin{array}{c} \psi (u_{q_{1}})\\ \vdots \\ \psi (u_{q_{|V\setminus I|}}) \end{array}}\right ]\!, \end{align*}

and

\begin{equation*} b_{1} = \left [{\begin{array}{c} 1\\ \vdots \\ 1 \end{array}}\right ]\!, \quad b_{2} = \left [{\begin{array}{c} 0\\ \vdots \\ 0 \end{array}}\right ]\!. \end{equation*}

Theorem 1: The system of linear equations given in Equation 3 has a single unique solution.

The proof of Theorem 1 can be found in Appendix A. As long as a linear equation system shown in Equation 3 can be obtained, some well known methods such as *Gaussian Elimination*, *Cramer’s Rule*, etc., can be used to solve it and we can get the infected score of each node under a certain infected node set $I$.

#### 3) Combined Score

Infecting score measures how a node infects others once it is infected, while infected score evaluates how possible this node will be infected with the current knowledge of the infected set $I$. Both factors should be taken into account when selecting the vaccinated nodes. Therefore, we combine them as follows to get a node $u$’s *combined score*.

\begin{equation*} \zeta (u,I) = \frac {\varphi (u,I)}{\max \limits _{v \in {V\setminus I}}\varphi (v,I)} \cdot \frac {\psi (u,I)}{\max \limits _{v \in {V\setminus I}}\psi (v,I)} \end{equation*}

At each round, the node with the highest combined score is selected as the candidate node. If this node is eligible for vaccination, it is removed from the graph and the combined score is recalculated based on the updated graph; if it has already been infected, it is added into set $I$ and the node with the highest combined score based on the updated $I$ is chosen as candidate. This process is repeated until $k$ nodes are selected for vaccination. Comparing with the adaptive algorithm in [20], our enhanced algorithm exploits the information of some known infected nodes during vaccination and combines both a node’s infecting score and infected score together to evaluate a node’s influence in disease propagation. The pseudo code of the enhanced algorithm is shown in Algorithm 1.

**Algorithm 1:**
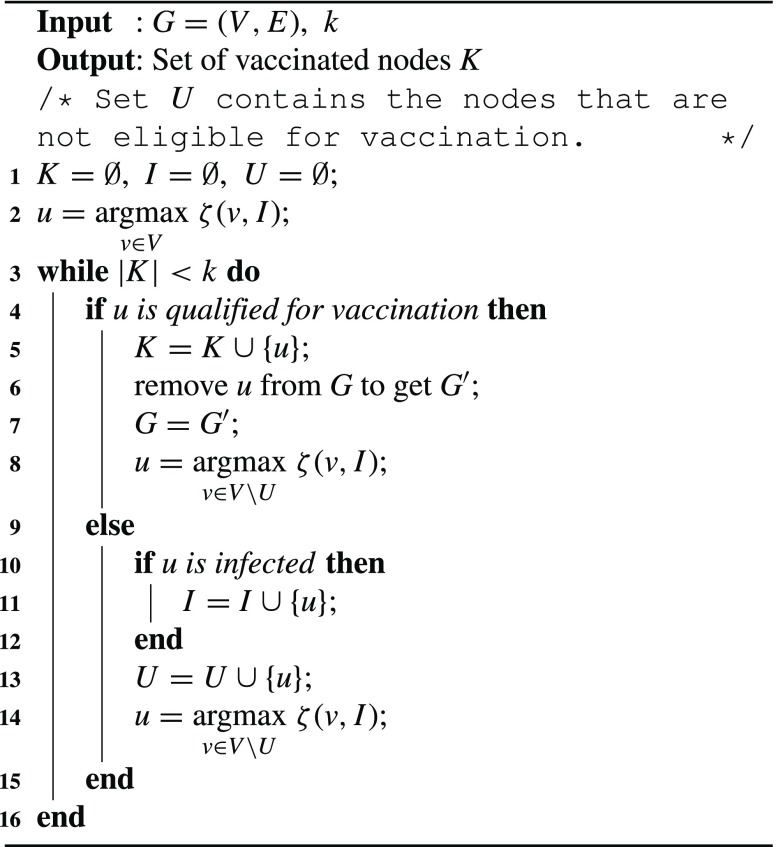
Enhanced Targeted Vaccination

## V. Performance Evaluations

In this section, we evaluate the performance of our centrality based algorithm and enhanced algorithm.

### A. Simulation Setup

The performance of our algorithms is evaluated based on the trace collected in the high school. The trace is divided into two halves based on the time when it was collected. We firstly use half of the trace as the training data to build the disease propagation graph, and use the other half for performance evaluations. Then we exchange the two halves and run the training and testing again for cross validation.

At the very beginning, we randomly choose a small group of nodes (1%) as the seed set of infection sources to initiate the infection process. The trace is executed based on time units. At each time unit (20 seconds), the SIR model [35] is used to simulate the infection process. In SIR, each node has three states: ${S}$ (Susceptible), ${I}$ (Infected) and ${R}$ (Recovered). For a node which is initially at state ${S}$, it will be infected with probability $\beta $ (called *transmission probability*) indoor and $\eta _{0}\beta $ ($\eta _{0}$ is set to 0.5) outdoor by contacting with an infected node. Once the node is infected, it will move into state ${I}$. An infected node may recover with a probability $\delta $ ($\delta $ is set to 0.0003) at each time unit and goes back to state ${R}$. Nodes in state ${R}$ will not get infected again since they have got immunization already.

Although some vaccination strategies have been proposed for certain diseases, e.g., ring vaccination for smallpox and targeted mop-up campaigns for polio, these strategies are based on the knowledge of infected nodes, which is not known in many scenarios. Therefore, instead of comparing with these strategies, we compare our centrality based algorithms and enhanced algorithm (*Enhanced*) with the community based scheme (*AFOCS*) [11] and the cluster based scheme (*Cluster*) [8]. Degree centrality, betweenness centrality and connectivity centrality are used to implement centrality based algorithm (denoted as *Degree, Betweenness* and *Connectivity* respectively). Closeness centrality is not used here since it does not work when the graph is disconnected.

*Vaccinating Threshold* $\alpha $ is used to control when the targeted vaccination starts. It is measured as the percentage of infected nodes in the network. This parameter represents the time delay since the infectious disease starts propagating till a vaccine is generated. Once the percentage of infected nodes reaches the threshold $\alpha $, we start to distribute vaccines to the selected nodes.

### B. Comparisons of Infection Rates

**FIGURE 7.**
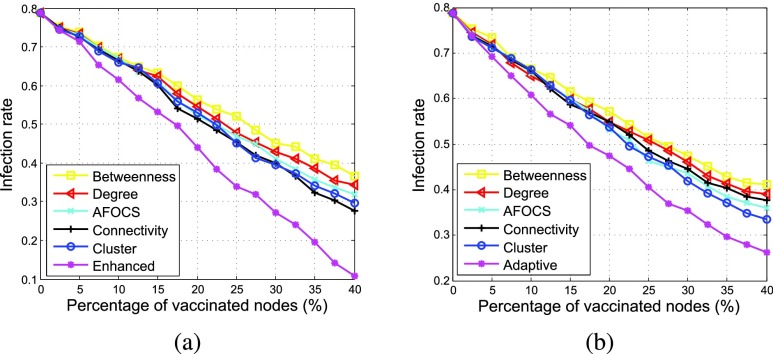
Effect of vaccinating threshold $\alpha $ ($\beta = 0.003$). (a) $\alpha = 2.5$%. (b) $\alpha = 10$%.

Figure 7 shows how the infection rate changes when the percentage of vaccinated nodes increases with $\alpha = \,\, 2.5$% and 10% respectively. As shown in the figure, no matter which scheme is used, the number of infected nodes will decrease with more vaccines distributed. *Enhanced* achieves better performance than other schemes under different $\alpha $. When $\alpha = \,\, 2.5$% and 20% of nodes are vaccinated, the infection rate of *Enhanced* is about 45%, but the infection rate of other schemes are higher than 50%. For the centrality based algorithms, under different $\alpha $, *Degree* performs better than *Betweenness* since disease is easier to transmit from the infected nodes to their neighbors than to those far away. *Connectivity* performs better than *Betweenness* and *Degree*, verifying that connectivity centrality is better to measure node’s importance for disease propagation.

Comparing Figure 7a with Figure 7b, we can see that *AFOCS* and *Cluster* perform worse than *Connectivity* when $\alpha = \,\, 2.5$%, but better when $\alpha = \,\, 10$%. The reason is as follows. If more nodes are infected before vaccination (i.e., $\alpha $ is larger), these infected nodes are more likely to be clustered together around the infected nodes. Since *AFOCS* and *Cluster* contain the disease by isolating infected communities or clusters, they can perform better when $\alpha $ is larger. However, their infection rate is still much higher than *Enhanced*.

### C. Infection Rate vs. Time

**FIGURE 8.**
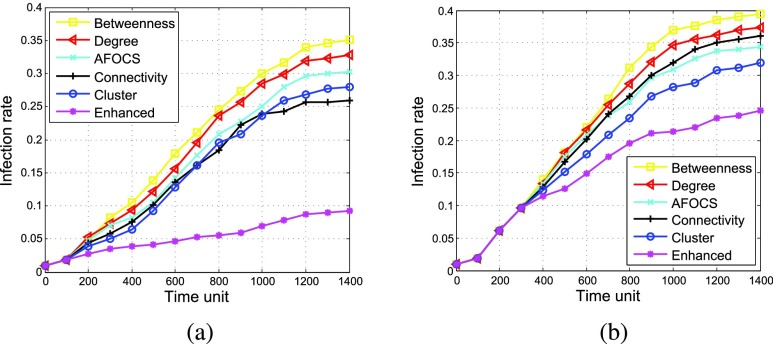
Infection rate vs. time (percentage of vaccinated nodes = 40%, $\beta = 0.003$). (a) $\alpha = 2.5$%. (b) $\alpha = 10$%.

Figure 8 shows how the infection rate changes over time with $\alpha = \,\, 2.5$% and 10%, respectively. The spread of the disease can be divided into three phases. At the beginning, the disease is slowly spread from the infection sources. Then, it propagates widely and the infection rate increases quickly. Finally, no more nodes will get infected and the infection rate keeps stable. Comparing with other schemes, *Enhanced* performs better as the infection rate increases more slowly and is bounded under a much lower level.

### D. Effect of Transmission Probability

**FIGURE 9.**
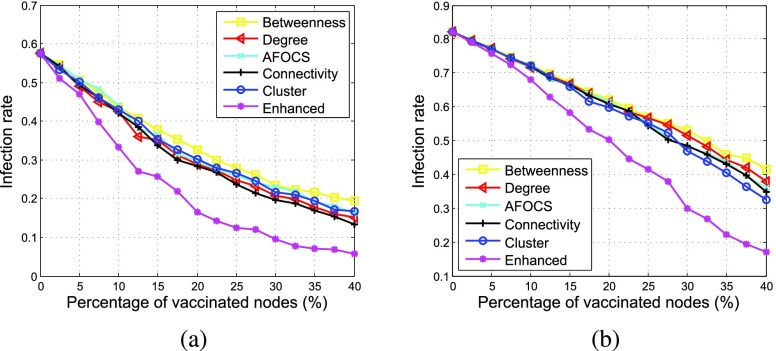
Effect of the disease transmission probability $\beta $ ($\alpha = 2.5$%). (a) $\beta = 0.002$. (b) $\beta = 0.004$.

Figure 9 shows how the disease transmission probability $\beta $ affects the spread of disease. As can be seen, *Enhanced* outperforms other schemes under different $\beta $. For centrality based algorithms, *Connectivity* achieves better performance than *Degree* and *Betweenness*. Comparing centrality based algorithms with *Cluster*, both *Connectivity* and *Degree* perform better than *Cluster* when $\beta = \,\, 0.002$, but *Cluster* performs better than *Connectivity* and *Degree* when $\beta = \,\, 0.004$. The reason is as follows. Generally speaking, if the infected nodes are uniformly distributed, centrality based algorithms will perform better; if the infected nodes are clustered together, *Cluster* will perform better. With a lower $\beta $, nodes will be infected more randomly, and then their distribution looks more uniform. With a higher $\beta $, nodes with close connections will be infected more easily and thus the infected nodes are more likely to be clustered together.

### E. Effect of Node Willingness

**FIGURE 10.**
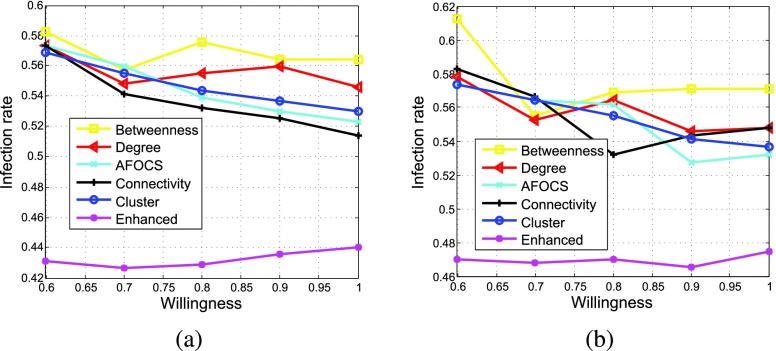
Effect of node willingness (percentage of vaccinated nodes = 20%, $\beta = 0.003$). (a) $\alpha = 2.5$%. (b) $\alpha = 10$%.

Because of the concerns on the potential side effects, not all nodes are willing to be vaccinated even if they are highly suggested. Thus, we assume each node is willing to be vaccinated with the same probability (called *willingness*). Figure 10 shows how node willingness affects the disease spread when different schemes are used under different $\alpha $. Comparing to other schemes, *Enhanced* is much more robust when node willingness varies. This is because the vaccinated nodes are adaptively chosen at each round in *Enhanced*. Even if a node is not willing to be vaccinated, its influence on the disease propagation is considered when selecting the next vaccinated nodes. However, in other schemes, the vaccinated nodes are calculated beforehand and node willingness is not considered. For *AFOCS* and *Cluster*, if certain bridge nodes (the nodes which connect different communities or clusters) are unwilling to be vaccinated, the goal for isolating the infected communities or clusters may fail.

### F. Performance in Scale-Free Networks

**FIGURE 11.**
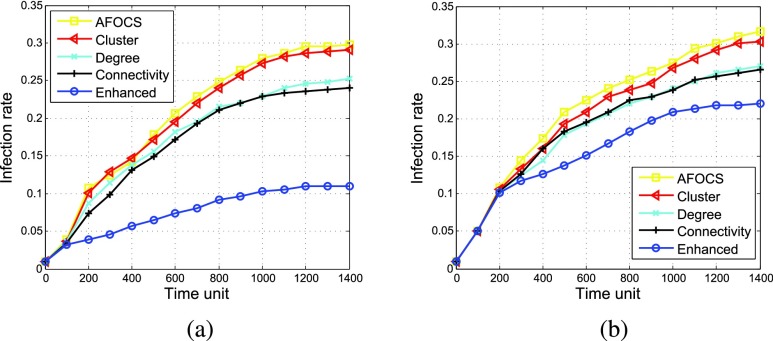
Infection rate vs. time in scale-free network (percentage of vaccinated nodes = 40%, $\beta = 0.003$). (a) $\alpha = 2.5$%. (b) $\alpha = 10$%.

In some social networks, the number of nodes’ neighbors follows power law distribution and the networks are scale free. In order to evaluate the performance of our algorithms in these networks, we generate a synthetic scale-free graph using Barabasi-Albert model [36]. Then we generate contacts in 1400 time steps based on the topology of this graph. At each time step, a node generates contacts with each neighbor with probability $p$, where $p$ is set to 0.3 in our simulation. With the synthetic contact trace, we can initiate the infection process and compare our enhanced algorithm with other algorithms. As shown in Figure 11, even in the scale-free network, *Enhanced* achieves better performance than other schemes. Comparing with *AFOCS* and *cluster*, centrality based algorithms perform better because they vaccinate the nodes with more neighbors first and these nodes are more likely to be infected in scale-free networks.

## VI. Conclusion

In this paper, we deployed a wireless sensor system in a high school to collect contacts happened between two students when they are within the disease transmission distance. Based on the collected traces, we construct a disease propagation graph to model the disease propagation, and propose a new metric called connectivity centrality to find the important nodes in the constructed graph for disease containment. Different from centrality measures like degree, betweenness or closeness centrality, connectivity centrality considers both a node’s local and global effect to measure its importance in disease propagation. Centrality based algorithms are presented and further enhanced by exploiting the information of the known infected nodes which can be detected during vaccination. We evaluate our algorithms and compare them with other schemes based on the real and synthetic traces. Simulation results show that our algorithms can contain infectious diseases effectively and outperform other schemes under various conditions.

## Appendix

Proof of *Theorem 1*: In Equation 3, since

\begin{align*} X_{1} = \left [{\begin{array}{c} \psi (u_{p_{1}})\\[2pt] \vdots \\[2pt] \psi (u_{p_{|I|}}) \end{array}}\right ] = \left [{\begin{array}{c} 1 \\[2pt] \vdots \\[2pt] 1 \end{array}}\right ]\!, \end{align*}

if we replace $w'(u_{q_{i}}, u_{q_{j}})$ with $a_{ij}$ for simplicity, Equation 3 can be rewritten as

\begin{align*}& A_{1}' X_{1} = b_{1}' \\[1.5pt]{}\smash {\left \{{\vphantom {\begin{matrix} \textstyle .\\\textstyle .\\\textstyle . \end{matrix}}}\right .}&\notag \\[-0.5em]& A_{2}' X_{2} = b_{2}' \end{align*}

where $A_{1}' = I_{|I| \times |I|}$, $b_{1}' = b_{1}$,

\begin{align*} A_{2}' = \left [{\!\begin{array}{ccccc} 1 & -a_{21} & -a_{31} & \cdots & -a_{|V\setminus I|1}\\[2pt] -a_{12} & 1 & -a_{32} & \cdots & -a_{|V\setminus I|2}\\[2pt] -a_{13} & -a_{23} & 1 & \cdots & -a_{|V\setminus I|3}\\[2pt] \vdots & \vdots & \vdots & \vdots & \vdots \\[2pt] -a_{1|V\setminus I|} & -a_{2|V\setminus I|} & -a_{3|V\setminus I|} & \cdots & 1\\[2pt] \end{array}\!}\right ] \end{align*}

and

\begin{align*} b_{2} = \left [{\begin{array}{c} \sum _{i=1}^{|I|} w'(u_{p_{i}}, u_{q_{1}})\\[2pt] \vdots \\[2pt] \sum _{i=1}^{|I|} w'(u_{p_{i}}, u_{q_{|V\setminus I|}})\\[2pt] \end{array}}\right ]\!. \end{align*}

Thus, to prove that the system of linear equations given in Equation 3 has a single unique solution, we only need to prove that Equation 5 has a single unique solution. Let $z = [z_{1}, z_{2}, {\dots }, z_{|V\setminus I|}]^\top $ denote a non-zero column vector of $|V\setminus I|$ real numbers and let $z^\top $ denote the transpose of $z$, then we have

\begin{align*} z^\top A_{2}' z=&z_{1} (z_{1} - a_{21} z_{2} - a_{31} z_{3} - {\dots }-a_{|V\setminus I|1} z_{|V\setminus I|})\\&+\, z_{2} (- a_{12} z_{1} + z_{2} - a_{32} z_{3} - {\dots }-a_{|V\setminus I|2} z_{|V\setminus I|}) \\&+\, {\dots }\\&+\, z_{|V\setminus I|} (- a_{1|V\setminus I|} z_{1} - a_{2|V\setminus I|} z_{3} - {\dots }+ z_{|V\setminus I|}) \end{align*}

Considering that $a_{ij} = a_{ji}$, the above equation can be rewritten as

\begin{equation*} z^\top A_{2}' z = \sum _{i = 1}^{|V\setminus I|} {z_{i}}^{2} - \sum _{i = 1}^{|V\setminus I|} {\sum _{j = 1, j > i}^{|V\setminus I|} (2 a_{ij} z_{i}z_{j})} \end{equation*}

Since ${(\sqrt {a_{ij}} z_{i} - \sqrt {a_{ij}} z_{j})}^{2} \geq 0$, i.e., $2 a_{ij} z_{i} z_{j} \leq a_{ij}{z_{i}}^{2} + a_{ij}{z_{j}}^{2}$, we have

\begin{align*} \sum _{i=1}^{|V\setminus I|}{\sum _{j=1, j>i}^{|V\setminus I|}}(2 a_{ij} z_{i} z_{j})\leq&\sum _{i=1}^{|V\setminus I|}{\sum _{j=1, j>i}^{|V\setminus I|}}(a_{ij}{z_{i}}^{2} + a_{ij}{z_{j}}^{2})\\=&\sum _{i=1}^{|V\setminus I|}{ \left[{\left({\sum _{j=1, j\neq i}^{|V\setminus I|} a_{ij}}\right) z_{i}^{2}}\right]} \end{align*}

and equality holds only when $z_{1} = z_{2} = \cdots = z_{|V\setminus I|} \neq 0$.

Since $a_{ij}$ is used to denote $w'(u_{q_{i}}, u_{q_{j}})$, $\sum _{j=1, j\neq i}^{|V\setminus I|} a_{ij} \leq 1$ and equality cannot hold for every $i$. Thus, for any non-zero column vector $z$, we have

\begin{align*} z^\top A_{2}' z=&\sum _{i = 1}^{|V\setminus I|} {z_{i}}^{2} - \sum _{i = 1}^{|V\setminus I|} {\sum _{j = 1, j > i}^{|V\setminus I|} (2 a_{ij} z_{i}z_{j})} \\\geq&\sum _{i = 1}^{|V\setminus I|} \left[{\left({1-\sum _{j=1, j\neq i}^{|V\setminus I|} a_{ij}}\right){z_{i}}^{2}}\right]\\>&0 \end{align*}

Therefore, symmetric matrix $A_{2}'$ is positive definite and $X_{2}$ in Equation 5 can be uniquely solved. Since $X_{1}$ in Equation 4 can also be uniquely solved, we prove that the system of linear equations given in Equation 3 has a single unique solution.
